# MyosinVIIa Interacts with Twinfilin-2 at the Tips of Mechanosensory Stereocilia in the Inner Ear

**DOI:** 10.1371/journal.pone.0007097

**Published:** 2009-09-23

**Authors:** Agnieszka K. Rzadzinska, Elisa M. Nevalainen, Haydn M. Prosser, Pekka Lappalainen, Karen P. Steel

**Affiliations:** 1 The Wellcome Trust Sanger Institute, Wellcome Trust Genome Campus, Hinxton, Cambridge, United Kingdom; 2 Institute of Biotechnology, University of Helsinki, Helsinki, Finland; University of Massachusetts Amherst, United States of America

## Abstract

In vertebrates hearing is dependent upon the microvilli-like mechanosensory stereocilia and their length gradation. The staircase-like organization of the stereocilia bundle is dynamically maintained by variable actin turnover rates. Two unconventional myosins were previously implicated in stereocilia length regulation but the mechanisms of their action remain unknown. MyosinXVa is expressed in stereocilia tips at levels proportional to stereocilia length and its absence produces staircase-like bundles of very short stereocilia. MyosinVIIa localizes to the tips of the shorter stereocilia within bundles, and when absent, the stereocilia are abnormally long. We show here that myosinVIIa interacts with twinfilin-2, an actin binding protein, which inhibits actin polymerization at the barbed end of the filament, and that twinfilin localization in stereocilia overlaps with myosinVIIa. Exogenous expression of myosinVIIa in fibroblasts results in a reduced number of filopodia and promotes accumulation of twinfilin-2 at the filopodia tips. We hypothesize that the newly described interaction between myosinVIIa and twinfilin-2 is responsible for the establishment and maintenance of slower rates of actin turnover in shorter stereocilia, and that interplay between complexes of myosinVIIa/twinfilin-2 and myosinXVa/whirlin is responsible for stereocilia length gradation within the bundle staircase.

## Introduction

Hair cells are specialized epithelial cells equipped with bundles of actin filled, microvilli-like cellular projections called stereocilia, that transform mechanical sound signals into electrical ones and enable the sensation of hearing to occur. The rows of stereocilia form staircase-like bundles that are deflected by sound waves thus increasing the tension on interstereocilial links and activating transduction channels [1]. The stereocilia length gradation and overall shape of the hair bundle are critical for hair cell frequency selectivity and its sensitivity. The actin filaments in the stereocilium core are uniformly polarized such that their barbed (plus, polymerizing) ends are located at stereocilia tips. The actin cores of stereocilia undergo continuous turnover at a rate proportional to stereocilia length. The polymerization of stereocilial actin starts at the tips and follows a treadmilling mechanism so that the organization of stereocilia bundles is dynamically maintained [2]. It remains unclear how a single cell can regulate differential actin renewal rates between rows of stereocilia.

Two of the unconventional myosins present in stereocilia show interesting distributions within bundles. The levels of myosinXVa at the stereocilia tips are correlated with stereocilia lengths from the early embryonic stages of bundle development [2]. In the absence of myosinXVa or its interacting partner whirlin, all stereocilia are very short and exhibit reduced gradation in stereocilia heights within each bundle [3]–[5]. In adult wild type stereocilia myosinVIIa is enriched at the tips from the second and shorter rows only and when myosinVIIa is absent the stereocilia are abnormally long [5]. In whirlin and myosinXVa mutant mice myosinVIIa is present in the tips of all stereocilia, which indicates that the stereocilia tip localization of myosinVIIa and whirlin/myosinXVa is mutually exclusive [5]. Mice that are homozygous mutants for both myosinXVa and myosinVIIa (*Myo15a^sh2/sh2^*, *Myo7a^4626SB/4626SB^*) exhibit disorganized and abnormally short stereocilia, which indicates that the effect of the lack of postnatal stereocilia elongation mediated by myosinXVa/whirlin complex dominates over lack of inhibition of stereocilia elongation mediated by myosinVIIa [6].

It is highly likely that the interaction between myosins and actin binding proteins participate in stereocilia length regulation. The list of major regulators of actin cytoskeleton includes proteins from the cofilin, profilin and twinfilin families. Cofilin is known to sever and depolymerise actin filaments at their pointed ends, whereas profilin inhibits spontaneous actin filament nucleation but also converts ADP-actin monomers to polymerization-competent ATP-monomers and localizes them to the sites of rapid filament assembly [7]–[9]. Twinfilin is an evolutionarily conserved protein, which inhibits actin filament assembly by sequestering actin monomers and by capping filament barbed ends [10]. In addition to actin, twinfilin binds heterodimeric capping protein and, at least in budding yeast, this interaction is necessary for twinfilin's correct sub-cellular localization [11]. Inactivation of twinfilin results in defects in cortical actin patches in budding yeast and in abnormal bristle morphology in *Drosophila* due to uncontrolled polymerization of actin [12], [13]. In mammals there are three isoforms of twinfilin, twinfilin-1, twinfilin-2a and twinfilin-2b. Twinfilin-2a and -2b are generated from the same gene through alternative promoter usage and differ only at the N-terminal region. Twinfilin-1 and twinfilin-2a/b share ∼65% sequence identity and the residues shown to be important for actin binding are highly conserved [14], [15]. Twinfilin-1 is the major isoform during development and in adult non-muscle cells, twinfilin-2a is expressed almost ubiquitously but at lower levels than twinfilin-1 and both twinfilin-1 and twinfilin-2a are highly expressed in hair cells. Twinfilin-2b is the predominant isoform of heart and skeletal muscles and appears to be expressed only in these tissues. Although the two mammalian twinfilin isoforms have distinct expression patterns their affinities for actin monomers or capping protein are comparable [15], [16]. The structure of twinfilin-1 has been solved recently [10], [17]. The structures of twinfilin-2a and -2b have not yet been solved, but they are expected to be similar to twinfilin-1.

Here we show that twinfilin localization at the tips of shorter stereocilia within the bundle staircase coincides with the myosinVIIa expression pattern and that twinfilin-2 interacts with myosinVIIa *in vivo* and *in vitro*. Exogenous myosinVIIa expression in fibroblasts negatively affects number of filopodia and encourages accumulation of twinfilin-2 at the filopodia tips. Our results suggest that the interaction between myosinVIIa and the actin-binding protein twinfilin may regulate stereocilia length within the bundle staircase.

## Results

### Twinfilin localizes to tips of shorter stereocilia in the presence of myosinVIIa

**Figure 1:**
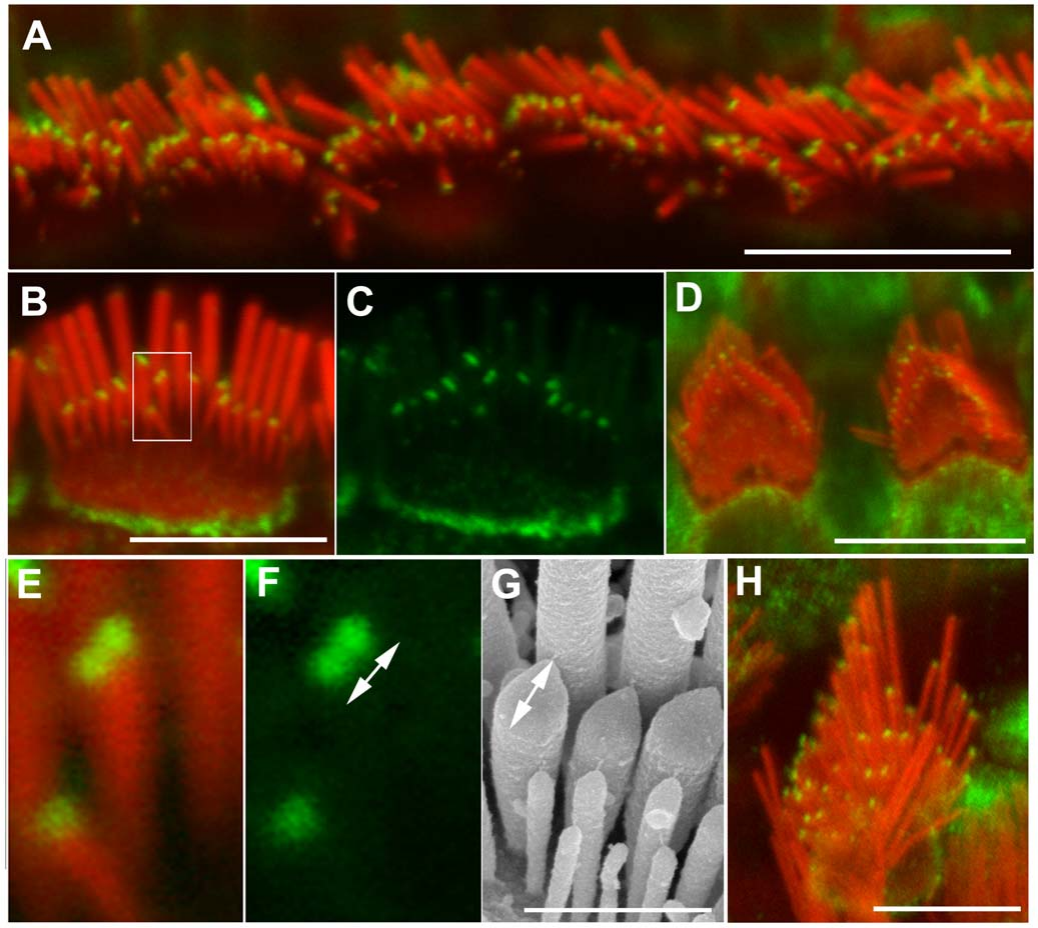
Pan-twinfilin localizes to the tips of shorter stereocilia. Confocal images showing the distribution of pan-twinfilin in stereocilia bundles. Actin filaments were counterstained with rhodamine/phalloidin (red). A–F and H–Pan-twinfilin (green) localizes to tips of shorter stereocilia of inner (A–C, E–F), outer (D) and vestibular (H) hair cells of wild type adult mice at P40. E–F–magnified image of single stereocilium (from B) from the second row showing two distinct spots of pan-twinfilin staining on the surface of the tip. The length of the pan-twinfilin fluorescent spot (F, 470±70 nm) corresponds with the length of the tip measured on SEM image (G, 440±50 nm). F and G show different bundles in similar orientation. Scale bars: A, D, H–10 µm; B–C–5 µm, E–G - 1 µm.

The mRNAs of the two mammalian twinfilin isoforms, twinfilin-1 and twinfilin-2 are highly expressed in the inner ear hair cells [15], [16]. However, the subcellular localization and function of twinfilins in hair cells was not known. Using previously characterized antibodies [14] we found that twinfilin-1 immunofluorescence was evenly distributed along the length of adult wild type stereocilia at P40 (when stereocilia bundles are fully mature from a morphological and physiological point of view) (supplemental data Fig. S1A) while being undetectable in developing bundles up to P6 (when the stereocilia bundles just reach morphological maturation) (data not shown). Unfortunately none of the three available antibodies specific to twinfilin-2a/b worked on mouse tissues (data not shown) in immunofluorescence. However, the antibodies recognizing all twinfilin isoforms (pan-twinfilin antibody) showed a greater enhancement of pan-twinfilin-specific immunofluorescence within the flattened area of the pointed tips of shorter stereocilia of outer and inner auditory hair cells and vestibular hair cells (Fig. 1). Pan-twinfilin staining was present in the tips of the tallest (most lateral) stereocilia but visibly less abundant than in shorter stereocilia on samples showing entire bundles and samples in which only the most lateral stereocilia were visible (where bundles were bent towards shorter stereocilia). Pan-twinfilin staining enhancement at the tips of shorter stereocilia was absent at P2 (when stereocilia are both elongating and widening), weak at P7 (supplemental data Fig. S2) but strong and very clear in stereocilia of adult animals (Fig. 1A–C). In some inner hair cell stereocilia we were able to resolve two distinct spots of pan-twinfilin staining, each spot of about 160 nm diameter, which is at the resolution limit of standard fluorescence microscopes. Other tips showed a single elongated spot of twinfilin staining measuring approximately 470±70 nm (n = 57). We believe that the elongated spots are likely to represent the merging of two ∼200 nm dots that could not be resolved individually. The observed staining pattern with weakening of fluorescence signal between dots suggests that each dot consists of two foci with centres about 200 nm apart each of which is formed by the binding of multiple antibody molecules. The measurements of the objects whose sizes are close to the microscope resolution limit on fluorescent images is not definitive and the size of a spot will depend on fluorescence intensity and threshold chosen. Therefore, the dimensions of the immunoflourescent spots we describe should be treated as approximate. In order to establish if the two adjacent dots of twinfilin specific immunofluorescence that we observe are likely to localize within a single stereocilium tip, as opposed to within two different stereocilia, we measured the length of the tips (length of the tips = the long axis diameter of the ellipsoid that demarks the shape of the stereocilia in its “tented” state). We found that the average length of tips of shorter stereocilia is about 440±50 nm (n = 123). We therefore believe that the double spots of twinfilin staining, or the single elongated spots, localize to single tips of the shorter stereocilia (Fig. 1B and E–G). In addition the pan-twinfilin staining enhancement was also observed within pericuticular necklace region (Fig. 1).

**Figure 2:**
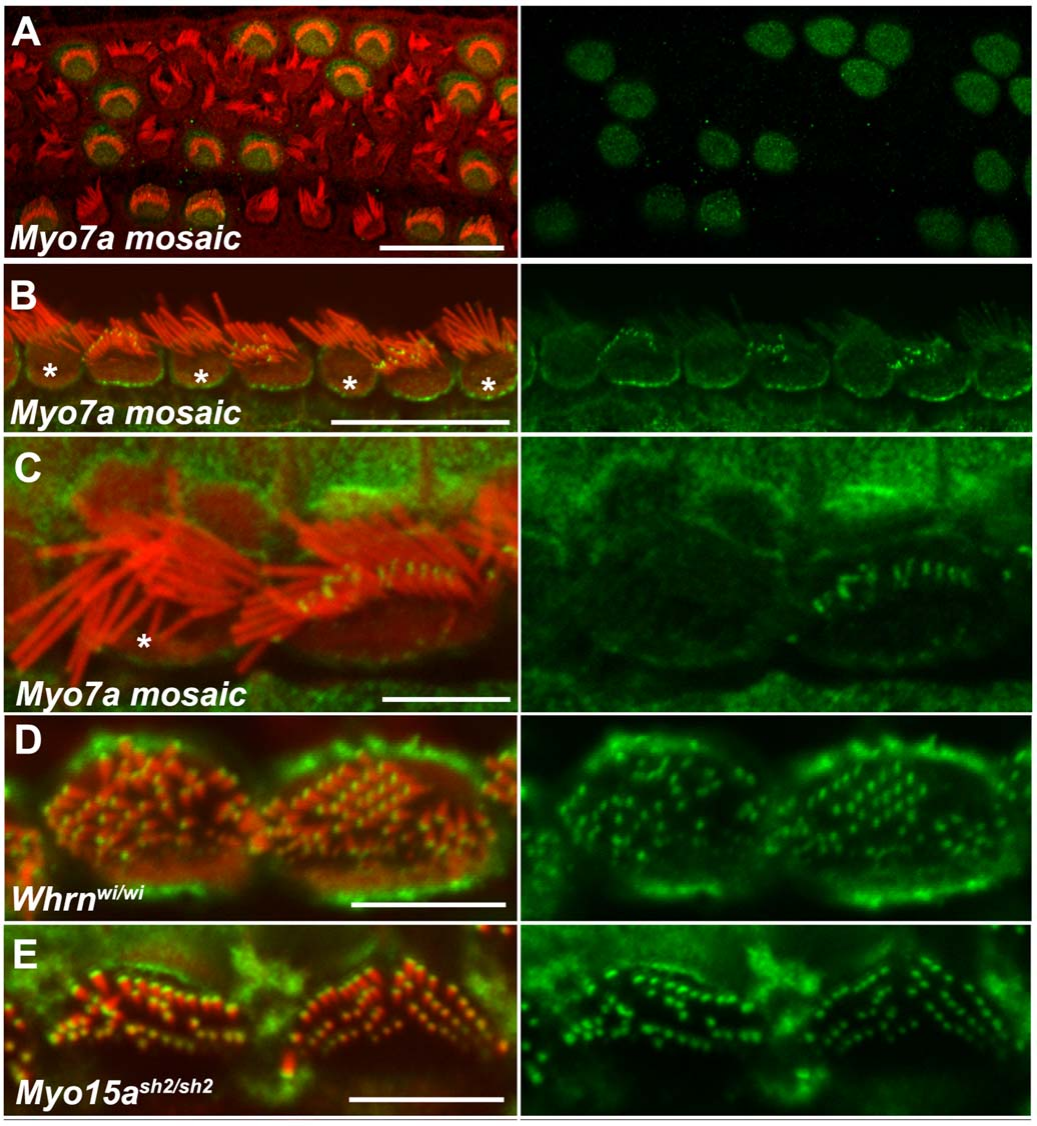
Myosin VIIa is necessary for pan-twinfilin localization at the stereocilia tips. Confocal images showing the distribution of pan-twinfilin in mutant stereocilia bundles. Actin filaments were counterstained with rhodamine/phalloidin (red). Images on the right show green channel. A - In mosaic auditory sensory epithelia of *Myo7a^4626SB/4626SB^ Hprt^(Myo7a)Brd/+^* females at P5 myosinVIIa immunofluorescence (green) is restricted to complemented hair cells with normal morphological appearance of stereocilia bundles. B, C - Pan-twinfilin immunofluorescence (green) at stereocilia tips is restricted to complemented inner hair cells (showing normal hair bundle morphology) while there is no staining in shorter stereocilia of non-complemented inner hair cells (asterisks). D–E - Pan-twinfilin (green) localized to the tips of all whirlin- and myosinXVa-deficient stereocilia on the apical surface of inner hair cells of adult *Whrn^wi/wi^* (D) and *Myo15a^sh2/sh2^* mice (E). Scale bars: A–10 µm; B–D and H–5 µm, E–G–1 µm.

The higher abundance of pan-twinfilin staining in shorter stereocilia rows versus the longest row indicated the possibility of twinfilin being involved in stereocilia length regulation. Therefore we analyzed twinfilin distribution in bundles formed by abnormally long stereocilia (myosinVIIa-null hair cells) and abnormally short stereocilia (hair cells lacking myosinXVa and/or whirlin). In order to analyze the relationship between the localization of twinfilin and myosinVIIa expression, we harvested auditory sensory epithelia from *Myo7a^4626SB/4626SB^Hprt^(Myo7a)Brd/+^* mosaic female mice at P40. Mosaic *Myo7a^4626SB/4626SB^Hprt^(Myo7a)Brd/+^* females are homozygotes for the shaker1 *Myo7a^4626SB^* allele (nonsense mutation, which reduces the protein to below detectable levels, [18] on chromosome7 and contain one copy of wild type *Myo7a* on the modified X-chromosome, which fully complements the shaker1 phenotype in approximately 50% of hair cells due to X-inactivation, providing an excellent *in situ* control [5] (Fig. 2A). As anti-myosinVIIa and anti pan-twinfilin antibodies are both rabbit polyclonal we were not able to indicate myosinVIIa-deficient hair cells by immunostaining or to evaluate co-localization of these two proteins. However, the characteristic morphological changes (smaller apical surface with bundles formed by fewer, disorganized and abnormally long stereocilia) allowed us to easily distinguish complemented and non-complemented (myosinVIIa-deficient) hair cells [5]. The pan-twinfilin staining was evident at the tips of shorter stereocilia from the second and subsequent rows on the apical surface of hair cells complemented with the expression of transgenic *Myo7a* (Fig. 2B,C), and its staining pattern was indistinguishable from that of wild type hair cells. However, enhanced pan-twinfilin staining was undetectable at the tips of myosinVIIa-deficient stereocilia (Fig. 2B,C) indicating that myosinVIIa is necessary for tip localization of twinfilin-2.

The enhancement of pan-twinfilin staining appeared to be more pronounced in the tips of wild type hair cells stereocilia from shorter rows. However all myosinVIIa-deficient stereocilia appeared abnormally long, which suggests that twinfilin-2 may also be co-localizing in wild type hair cells with myosin-VIIa at the tips of the longest stereocilia but at levels too low to be detected by immunofluorescence. In order to test if in the absence of myosinVIIa the shorter stereocilia show greater length increase we measured the length of stereocilia in both mutant and complemented inner hair cells of *Myo7a^4626SB/4626SB^Hprt^(Myo7a)Brd/+^* mosaic female mice at P28 using previously obtained SEM images [5] and compared stereocilia length between the most lateral and middle rows. The average length of stereocilia from the second row in wild type inner hair cells from the middle turn of the cochlear duct was 1.27±0.13 µm (n = 47) compared with 2.15±0.39 (n = 48) in myosinVIIa-deficient hair cells, which indicates that the stereocilia (second row) are about 70% longer in the absence of myosinVIIa. The average length of stereocilia from the most lateral (tallest) row was 2.07±0.43 (n = 378) for wild type and 3.02±0.57 (n = 323) for myosinVII-deficient hair cells indicating a stereocilia length increase of about 45%. Both measurements were performed on the same set of images).

Next we analyzed immunolocalization of pan-twinfilin in auditory epithelia of *Whrn^wi/wi^* and *Myo15a^ sh2/sh2^* mice as these mutants exhibit very short stereocilia and tips of all stereocilia within single bundles contain myosinVIIa [5]. Pan-twinfilin staining was enhanced at the tips of all stereocilia of *Whrn^wi/wi^* (Fig. 2D) and *Myo15a^ sh2/sh2^* (Fig. 2E) adult mice, and not just the shorter stereocilia. Twinfilin localization at the tips of all stereocilia of whirler mutants was observed early as postnatal day 7 (supplemental data Fig. S2). The distribution of pan-twinfilin labelling in *Whrn^+/wi^* and *Myo15a^+/sh2^* littermate control tissues was indistinguishable from wild type controls (data not shown). The pattern of twinfilin-2 immunoreactivity in mutants with abnormally short stereocilia was markedly similar to the distribution of myosinVIIa [5]. The intensity of twinfilin-1 staining was unaffected by the levels of whirlin or myosinVIIa (supplemental data Fig. S1).

### Twinfilin-2 interacts with myosinVIIa *in vivo* and *in vitro*

**Figure 3:**
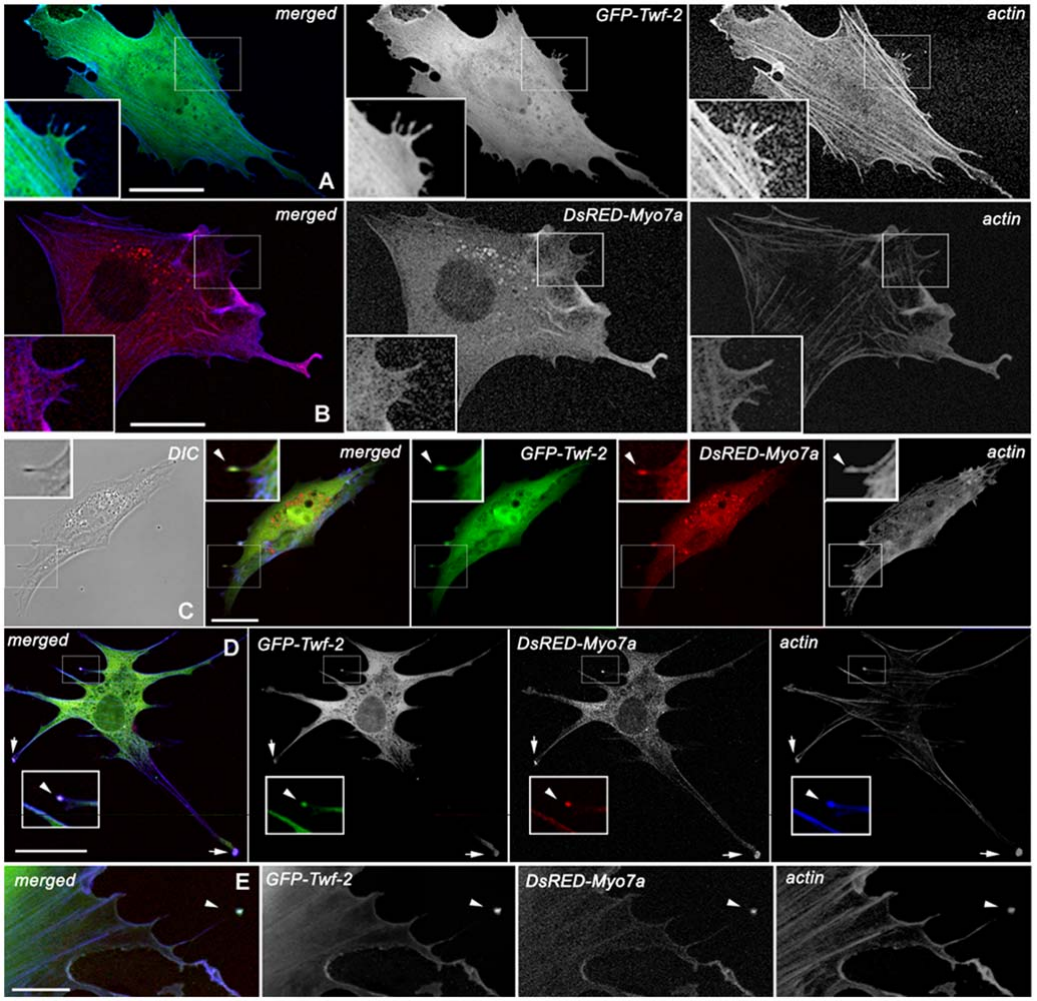
Wild type full-length DsRED-myosinVIIa (red) co-localizes with GFP- twinfilin-2 (green) in filopodia tips. Confocal images showing distribution of GFP-twinfilin-2, DsRED-myosinVIIa, in BHK-21 cells. Cortical actin was stained with AlexaFluor633/phalloidin (blue). A GFP-twinfilin-2 alone localizes predominantly along the filopodium length. B DsRED-myosinVIIa alone localizes predominantly along the filopodium length. C–E Co-transfection of *GFP-Twf-2* and *DsRED-Myo7a* reveals co-localization of co-expressed proteins at the filopodium tip (arrow heads) and adhesion plaques (arrows) in representative BHK-21 cells. Scale bars: A–H -25 µm.

**Figure 4:**
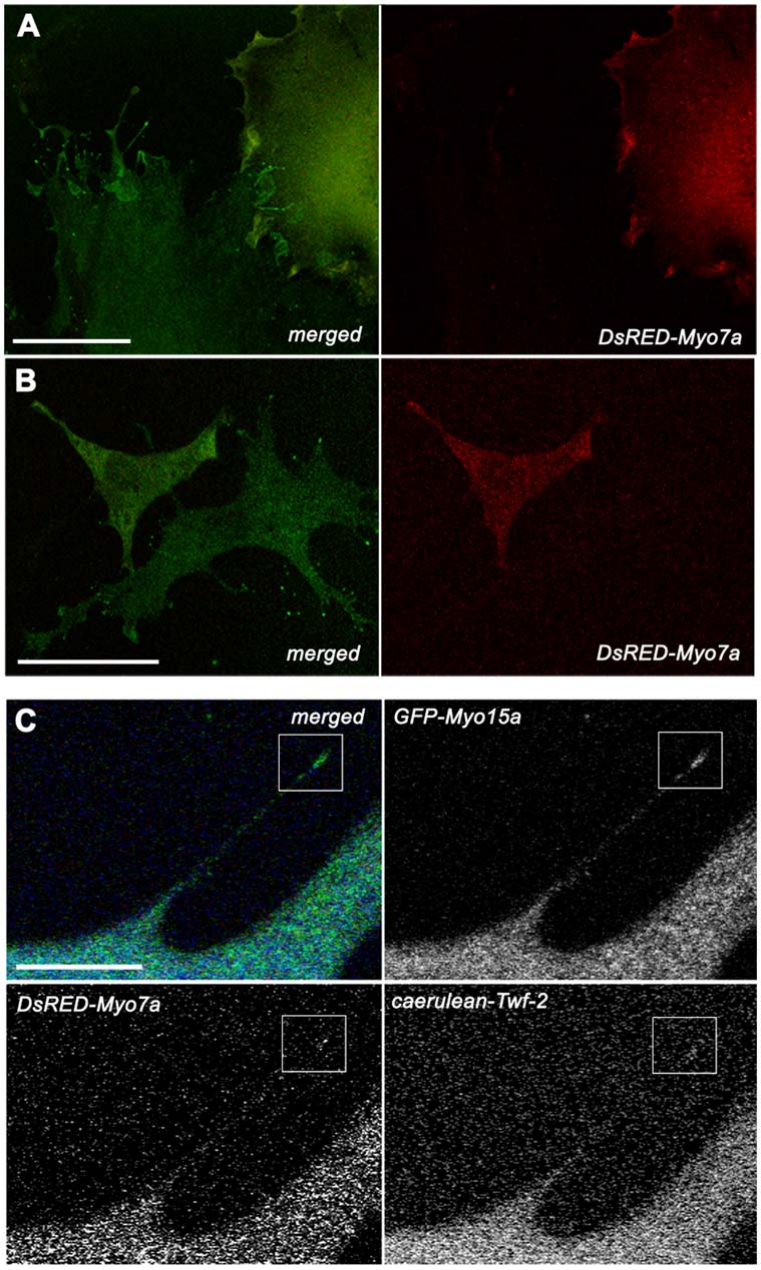
Levels of exogenous expression of *DsRED-Myo7a* correlate with lower number of filopodia in BHK-21 fibroblasts. A–B Higher levels of *DsRED-Myo7a* fluorescence in cell cytoplasm correlated negatively with number of filopodia in cells expressing *GFP-Myo15a*. In fibroblast showing low levels of *DsRED-Myo7a* fluorescence filopodia were numerous and *GFP-Myo15a* was clearly visible at their tips. C In cells co-transfected with *GFP-Myo15a*, *DsRED-Myo7a* and *caerulean-Twf-2* all three proteins localized to filopodia tips. Scale bars: A–B -50 µm, C–10 µm.

The similar subcellular distribution of twinfilin-2 and myosinVIIa in control and mutant hair cells suggested that these two proteins may interact with each other, however to date such an interaction has not been shown. We evaluated if twinfilin-2 and myosinVIIa co-localize when co-expressed by transfecting BHK-21 cells with the full-length expression constructs *GFP-Twf-2* and *DsRED-Myo7a*. When transfected alone, either twinfilin-2 (Fig. 3A) or myosinVIIa (Fig. 3B), localized along the entire length of the filopodium (n = 30 for *GFP-Twf-2* and n = 30 for *DsRED-Myo7a*). When co-transfected together myosinVIIa and twinfilin-2 (n = 30) co-localized at the tips of filopodia and within focal adhesion sites (Fig. 3C–E). We confirmed the presence of myosinVII and twinfilin-2 within focal adhesion sites by immunovisualizing vinculin in BHK-21 fibroblasts transfected with *cerulean-Twf-2* alone (n = 70) and *cerulean-Twf-2* and *DsRED-Myo7a* (n = 37) (supplemental data Fig. S3). Interestingly, when we co-transfected myosinVIIa and myosinXVa (n = 150) only myosinXVa localized to filopodia tips and the number of filopodia appeared to be reduced (Fig. 4A, B). This observation may indicate that myosinVIIa is likely to influence the elongation of parallel, uniformly polarized actin filaments in the filopodia cores.

Therefore we quantified this phenomenon by measuring pixel intensity for red (myosinVIIa) and green (myosinXVa) fluorescence in the cell cytoplasm and counting the number of filopodia on individual cells. In a pool of individually imaged cells (n = 66) we observed a negative, significant correlation between filopodia number and red-to-green pixel intensity ratio as well as between filopodia number and red pixel intensity (CC = −0.38, *t* = −3.25, p< = 0.0019 and CC = −0.37, *t* = −3.16, p = 0.0024 respectively; df = 64). The correlation between filopodia number and green pixel intensity was not significant (CC = −0.19, *t* = −1.56, p = 0.12, df = 64). In addition we performed paired comparisons using pairs of transfected cells (n = 29) in order to control for variation that could occur between frames. The difference in filopodia numbers was significant between paired data sets showing low and high red-to-green pixel intensity ratios (*t*_s_ = 4.13, p = 0.00029), highly significant between data sets showing low and high red pixel intensity (*t*_s_ = 5.72, p = 0.000006,) and not significant between data sets showing low and high green pixel intensity (*t*_s_ = 1.29, p = 0.207). Our results suggest that the amount of myosinVIIa in the cell cytoplasm correlates with the number of actin filled cellular protrusion. In addition we performed triple transfection experiments (n = 190) and in the presence of twinfilin-2 both myosins were present at the filopodia tips (Fig. 4C). These results indicate direct interaction between myosinVIIa and twinfilin-2.

**Figure 5:**
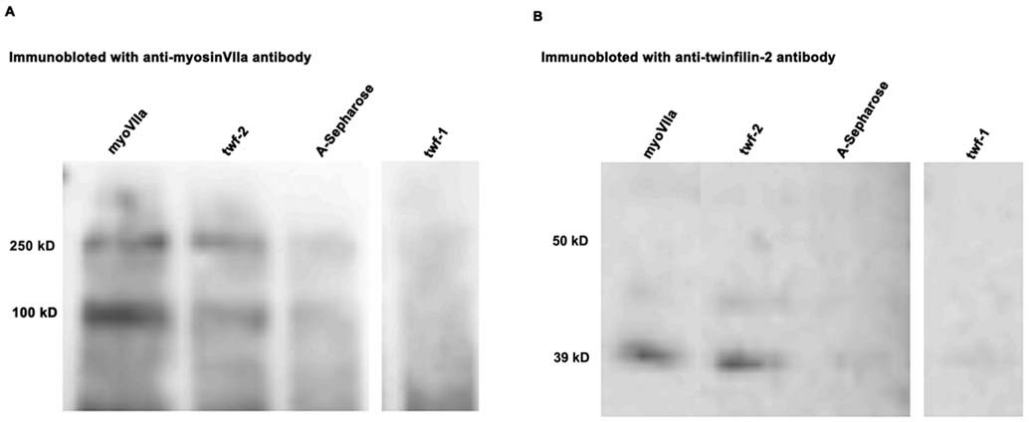
Twinfilin-2 interacts with myosinVIIa *in vivo*. The interaction between twinfilin-2 and myosinVIIa was demonstrated by immunoprecipitation from protein lysates of inner ear tissue with anti myosinVIIa antibody (myoVIIa), anti twinfilin-2 antibody (twf-2), protein A-Sepharose and anti twinfilin-1 (twf-1) antibody followed by the immunoblot with a anti-myosinVIIa antibody (A) and anti-twinfilin-2 antibody (B). Procedures for immunoprecipitations and Western blots are described under Materials and Methods (Supp. information). Twinfilin-2 has a molecular weight of 39 kD. MyosinVIIa has a molecular weight of about 250 kD; the band at 100 kD may be a result of protein degradation.

To further confirm the interaction of twinfilin-2 and myosin7a we performed co-immunoprecipitation experiments on lysates from freshly dissected inner ear sensory epithelia using antibodies against myosinVIIa, twinfilin-1 and twinfilin-2. The twinfilin-2 specific antibody had lost its activity for immunofluorescence studies, but could be used in co-immunoprecipitation experiments. The amount of myosinVIIa (MW ∼250 kD) co-immunoprecipitated with anti-twinfilin-2 antibody (Fig. 5A, twf-2) is clearly above background (Fig. 5A), whereas no detectable amounts of myosinVIIa co-immunoprecipitated when using anti-twinfilin-1 antibody (Fig. 5A, twf-1). The inverse experiment showed that twinfilin-2 (MW ∼39 kD) co-immunoprecipitated when using anti-myosinVIIa antibody (Fig. 5B, myoVIIa). Twinfilin-1 did not co-immunoprecipitate when using anti-myosinVIIa antibody (data not shown). The ∼100-kD band seen in myosinVIIa-antibody detected blots is likely to be a protein that binds non-specifically to protein A-Sepharose beads and cross-reacts with the myosinVIIa antiserum or alternatively a degradation product of myosinVIIa. Lower bands were previously observed by Hasson and colleagues with the same antibody [19] and appear on western blots for various tissues (data not shown). The co-immunoprecipitation data show that twinfilin-2 interacts with myosinVIIa in vivo but it remains unclear if the interaction is direct or if myosinVIIa is a part of a multiprotein complex, whereas twinfilin-1 does no interact with myosinVIIa.

## Discussion

Our findings suggest that myosinVIIa interacts with twinfilin-2, an actin polymerization inhibitor, which is differentially localized within the stereocilia bundle. Twinfilin-specific immunofluorescence forms two distinct spots at the barbed ends of actin filaments within the pointed tips of stereocilia from the second row (Fig. 1, supplemental data Fig. S2) and its distribution within stereocilia bundles correlates with distribution of myosinVIIa [5]. Overlapping distribution of myosinVIIa and twinfilin-2 immunolocalization was not previously reported in the literature. Twinfilin is first detectable at the tips of shorter stereocilia during postnatal development and the time of its appearance coincides with termination of postnatal stereocilia elongation [20], [21]. The absence of myosinVIIa influences stereocilia length but does not affect the tip localization of myosinXVa or whirlin in the abnormally long stereocilia of shaker1 mutant (myosinVIIa null allele) mice [5]. In contrast, twinfilin is absent from myosinVIIa-deficient stereocilia of shaker1 mice mutants. Lack of either myosinXVa or whirlin at the tips results in the formation of very short stereocilia arranged in a staircase-like bundle where stereocilia length gradation is significantly reduced [3], [4]. However, in the absence of myosinXVa and/or whirlin it is myosinVIIa [5] and twinfilin-2 that are present at all stereocilia tips of shaker2 and whirler mutant mice. Interestingly, in whirler mice the stereocilia elongate slightly during early postnatal development but a few days later they start to shorten [22] and the onset of shortening coincides with an appearance of twinfilin at the stereocilia tips. The mice homozygous for the *Myo15a^sh2/sh2^* and *Myo7a^4626SB/4626SB^* alleles, which presumably lack both myosin XVa/whirlin and myosinVIIa/twinfilin complexes show very short and disorganized stereocilia, which suggests that the effect of the lack of postnatal stereocilia elongation dominates over lack of inhibition of stereocilia elongation [6].

In co-precipitation experiments on inner ear epithelia lysates myosinVIIa was pulled down with anti-twinfilin-2 antibody and twinfilin-2 was pulled down with anti-myosinVIIa antibody while no co-precipitation was observed between myosinVIIa and twinfilin-1, indicating that there is a physical interaction between myosinVIIa and twinfilin-2. Twinfilin-1 and twinfilin-2 are very similar in sequence and biochemical function, but they are differentially regulated in cells and may have different roles in the regulation of the actin cytoskeleton dynamics in stereocilia [15], [16]. In co-expression experiments full length DsRED-myosinVIIa and GFP-twinfilin-2 co-localized at the tips of filopodia. Interestingly, the triple-transfection experiments revealed that in the presence of twinfilin-2 both myosins, VIIa and XVa co-localize at filopodia tips. The statistical analysis showed that higher levels of myosinVIIa in the cell cytoplasm strongly correlate with filopodia suppression.

Stereocilia start to develop around embryonic day 14.5 from bundles of microvilli of constant dimensions and with a kinocilium (modified primary cilium formed by microtubules) at the centre of the bundle. During development of the stereocilia bundles the kinocilium moves to the periphery, stereocilia lengthen and widen gradually until they reach a predetermined size and the excess stereocilia are resorbed. The stereocilia near the kinocilium start to elongate first and they finish elongation last, forming the tallest and the most lateral row. The stereocilia length gradation within the bundle staircase is established before the full length is reached and most of the growth of stereocilia occurs postnatally. MyosinXVa localizes to stereocilia tips and its immunostaining overlaps with the barbed ends of actin filaments in the stereocilia core from the earliest stages of their development [2], [23]. The levels of myosinXVa are directly proportional to stereocilia lengths, which indicate that myosinXVa may be involved in differential stereocilia elongation within bundle staircases [2]. MyosinXVa interacts with whirlin, which is present in stereocilia tips transiently during postnatal stereocilia elongation [24], [25]. In adult stereocilia, which have finished their elongation, whirlin expression is limited to the tips of the longest and most lateral stereocilia [26]. In mice homozygous for the shaker2 allele, myosinXVa and whirlin are absent from stereocilia tips, while in mice homozygous for the whirler allele no whirlin is detected at the stereocilia tips but tip localization of myosinXVa is not affected [24]. The stereocilia of shaker2 mutant mice lack any tip-density while in whirlin-deficient stereocilia tip-density is reduced to a few patches indicating that myosinXVa may form a scaffold, which is necessary for whirlin localization at the tip [2], [27].

The molecular scaffold at the stereocilia tips formed by myosinXVa and whirlin could enable more actin monomers increasing G-actin concentration at the barbed end and promoting actin filament polymerization. Twinfilin could either regulate the pool of available ATP-actin at the barbed ends or directly cap actin filaments at the tips of stereocilia from the second and shorter rows. By localizing its activity at the tips of stereocilia, twinfilin would not affect actin processes in other parts of the same cell, and so would not interfere with the cytoplasmic pool of available actin monomers. Twinfilin might also inhibit actin polymerization *via* its interaction with a capping protein, for example by localizing the capping protein to stereocilia tips [11], however we were not able to detect capping protein at the tips with available antibodies [28], [29] (data not shown). The precise nature of the interaction between myosinVIIa and twinfilin remains unclear, but myosinVIIa could i) transport twinfilin-2 to stereocilia tips, ii) anchor twinfilin-2 to the tips and/or iii) intensify the actin polymerization inhibitory properties of twinfilin-2.

Previous results indicate that the myosinXVa/whirlin complex promotes the polymerization of actin filaments at the barbed ends. Our current results strongly suggest that myosinVIIa forms a complex with twinfilin-2 and that this complex inhibits polymerization of actin in stereocilia cores. Therefore we propose that the balance between these two complexes determines the stereocilia length gradation within bundle staircases.

## Methods

### Ethics statement

All animal studies were licensed by the U.K. Home Office under the Animals (Scientific Procedures) Act 1986 and were approved by Wellcome Trust Sanger Institute Ethical Review Committee.

#### Immunohistochemistry

Pan-twinfilin, twinfilin-1 and myosinVIIa were immunolocalized in cochleae isolated from wild type (C3HeB/FeJ) mice at P2 (n = 3), P7 (n = 3) and P40 (n = 2), *Myo7a^4626SB/4626SB^ Hprt^(Myo7a)Brd/+^* females mosaic for myosinVIIa expression at P40 (n = 2), *Whrn^wi/wi^* (n = 2) and *Whrn^+/wi^* (n = 2) mice at P40, *Whrn^wi/wi^* (n = 2) and *Whrn^+/wi^* (n = 3) mice at P7 and *Myo15a^sh2/sh2^* (n = 1) and *Myo15a^+/sh2^* (n = 1) mice at P60. Tissues were fixed in 4% paraformaldehyde for 2 h at room temperature as previously described and exposed to rabbit polyclonal antibodies against pan-twinfilin (developed against mouse recombinant twinfilin), twinfilin-1 (twinfilin-2-binding fraction was removed from anti-pan-twinfilin antibody using a twinfilin-2 affinity column) [16] and myosinVIIa (Proteus BioSciences, Ramona, CA) (all diluted 1∶300), anti-rabbit AlexaFluor488 antibody (1∶1000) and rhodamine/phalloidin (1∶200). We also used hen anti-twinfilin-2 (with anti-mouse AlexaFluor488 secondary antibody) and anti-Ptk9l (developed against peptide, Abgent Europe, Switzerland) both diluted 1∶300 but neither one worked at the level of immunofluorescence. Samples were analysed with a LSM510Meta confocal microscope (Zeiss, Welwyn Garden City) using a 63x 1.4 n.a. objective. Post acquisition image analyses were performed using Adobe Photoshop CS2. Measurements of stereocilia and staining dimensions were performed using Image J software. Statistical analyses were performed with Xcel software.

#### Modification of *GFP-Myo7a* and *GFP-Twf-2* plasmids

MyosinVIIa was fluorescently tagged with DsRED Monomer (Clontech) by subcloning the *Myo7a* as an *EcoRI/SalI* restriction fragment from a *GFP-Myo7a* expression plasmid received from Dr. T. Friedman [24] into the DsREDC1 plasmid as an in-frame fusion. Prior to subcloning the *Myo7a* gene, the correct frame for the fusion was created by modifying the pDsREDMonomerC1 polylinker at the *XhoI/SalI* site by cloning the two annealed oligonucleotides as a double stranded DNA fragment: 5′TCGAGCCTCAAGCTTCGAATTCAAAAAA G 3′ and 3′CGGAGTTCGAAGCTTAAGTTTTTTCAGCT5′.

*GFP-Twf-2* was created by excising the *Twf-2* cDNA sequence from the plasmid pPL68 [16] with *SpeI-HindIII* and ligating it into pEGFP-C1A vector to create plasmid pPL193. *Caerulean-Twf-2* plasmid was created by subcloning the *Twf-2* as an *BspEI/HindIII* restriction fragment from a pPL193 expression plasmid into the caerulean plasmid (Addgene) as an in-frame fusion.

#### BHK-21 cell culture and transfections

Syrian hamster kidney cells (BHK-21) were cultured at 37°C and 5% CO_2_ in MEM supplemented with 10% fetal bovine serum. For transfection, cells were grown on a cover slip. Cells were transfected 24 h after seeding with *DsRED-Myo7a*, *GFP-Twf-2* and *GFP-Myo15a* expression constructs using Lipofectamine 2000 (Invitrogen). For triple transfection experiments fibroblasts were transfected *DsRED-Myo7a and GFP-Myo15a* at one time-point and 24 h later were transfected with *caerulean-Twf-2*. Cells were fixed 48 h after transfection with 4% paraformaldehyde and half of the cultures were stained with AlexaFluor633/phalloidin to visualize cortical actin. Triple transfected cultures were not counterstained. For vinculin immunostaining, cultures were transfected with *cerulean-Twf2* alone or co-transfected with *cerulean-Twf2* and *DsRED-Myo7a*. Transfected cultures were then fixed for 5 min with 4% paraformaldehyde, permeabilized for 2 min with 0.05% Triton X-100, blocked for 15 min with 4% BSA, incubated for 2 h with anti-vinculin rabbit polyclonal antibodies (Sigma, dilution 1∶50) and for 40 min with anti rabbit Alexa Fluor 488 secondary antibodies. Slides were analyzed with a LSM510Meta confocal microscope (Zeiss) using 40x NA 1.3 and 63x NA 1.4 objectives. Pixel intensity analyses were performed using Image J software and background was subtracted. Statistical analyses were performed with Xcel software using correlation coefficient and t-test for the paired comparisons.

#### Co-immunoprecipitation experiments

Co-IPs were performed on lysate obtained from freshly isolated inner ear sensory epithelia (auditory epithelia and maculae utricule and saccule) of wild type mice at P28 (n = 24). Tissue was lysed in buffer containing 50 mM Tris-HCl, pH 7.0, 150 mM NaCl, 1% Triton-X 100, 2 mM EDTA, 0.2 mM PMSF and Complete Protein Inhibitor Cocktail (Roche). The lysates were precleared with 5% protein A-Sepharose (GE Healthcare) in the lysis buffer for 2 h at 4°C and incubated overnight at 4°C with only 5% protein A-Sepharose or with anti-myosinVIIa, rabbit polyclonal anti-twinfilin-1, hen anti-twinfilin-2 (developed against his-tagged twinfilin-2) [16] and 5% protein A-Sepharose in lysis buffer. The precipitates were washed four times with the same buffer and subjected to SDS-PAGE. Western blotting was carried out as described previously with the following primary antibodies: anti-myosinVIIa (1∶1000) (Proteus BioSciences, Ramona, CA), anti-twinfilin-1 (1∶500) [5] or anti-twinfilin-2 antibody (1∶500) and secondary antibodies: alkaline phosphatase-conjugated anti-rabbit antibody (1∶10,000, Promega), or with alkaline phosphatase-conjugated anti-hen antibody (1∶10,000, Promega).

## Supporting Information

Figure S1 Twinfilin-1 localizes along the length of stereocilia and its immunostaining is not affected by the lack of myosin VIIa or whirlin. Confocal images showing the distribution of twinfilin-1 (green) in stereocilia bundles on the apical surface of inner hair cells. Actin filaments were counterstained with rhodamine/phalloidin. Images on the right show green channel. (A) Wildtype adult mouse, (B) Whrn+/wi adult mouse, (C) Whrnwi/wi adult mouse, (D) The mosaic epithelia of an adult Myo7a4626SB/4626SB Hprt(Myo7a)Brd/+ female (asterisks indicate non-complemented, myosinVIIa-deficient cells). Scale bars: A–D 10 µm.
(10.98 MB TIF)

Figure S2 Twinfilin appears in the stereocilia tips between Postnatal Day (PD) 2 and 7 Confocal images showing no pan-twinfilin staining (green) at the tips of control hair cells at PD2 (A). At PD7 pan-twinfilin staining was present at the tips of shorter stereocilia in wild type hair cells (B) and in tips of all stereocilia of Whrnwi/wi mice (C). Scale bars: A 10 µm, B–C 5 µm.
(3.21 MB TIF)

Figure S3 Cerulean-Twf2 and DsRED-Myo7a co-localize with vinculin within focal attachment sites. Confocal images showing BHK-21 fibroblasts transfected with cerulaean-Twf2 alone (A) and co-transfected with cerulean-Twf2 and DsRED-Myo7a (B) and stained with anti-vinculin antibodies. Cerulean-Twf2 and DsRED-Myo7a localize to focal adhesion sites visualized by anti-vinculin immunolabeling. However, in all double transfected cells the Cerulaean-Twf2 signal is very weak and diffuse. Scale bars A,B 20 µm
(9.32 MB TIF)
