# Supplementary figures and images for: MyosinVIIa Interacts with Twinfilin-2 at the Tips of Mechanosensory Stereocilia in the Inner Ear
Source: PLoS One. 2009 Sep 23;4(9):e7097. doi: 10.1371/journal.pone.0007097 (PMC2743196; doi:10.1371/journal.pone.0007097)

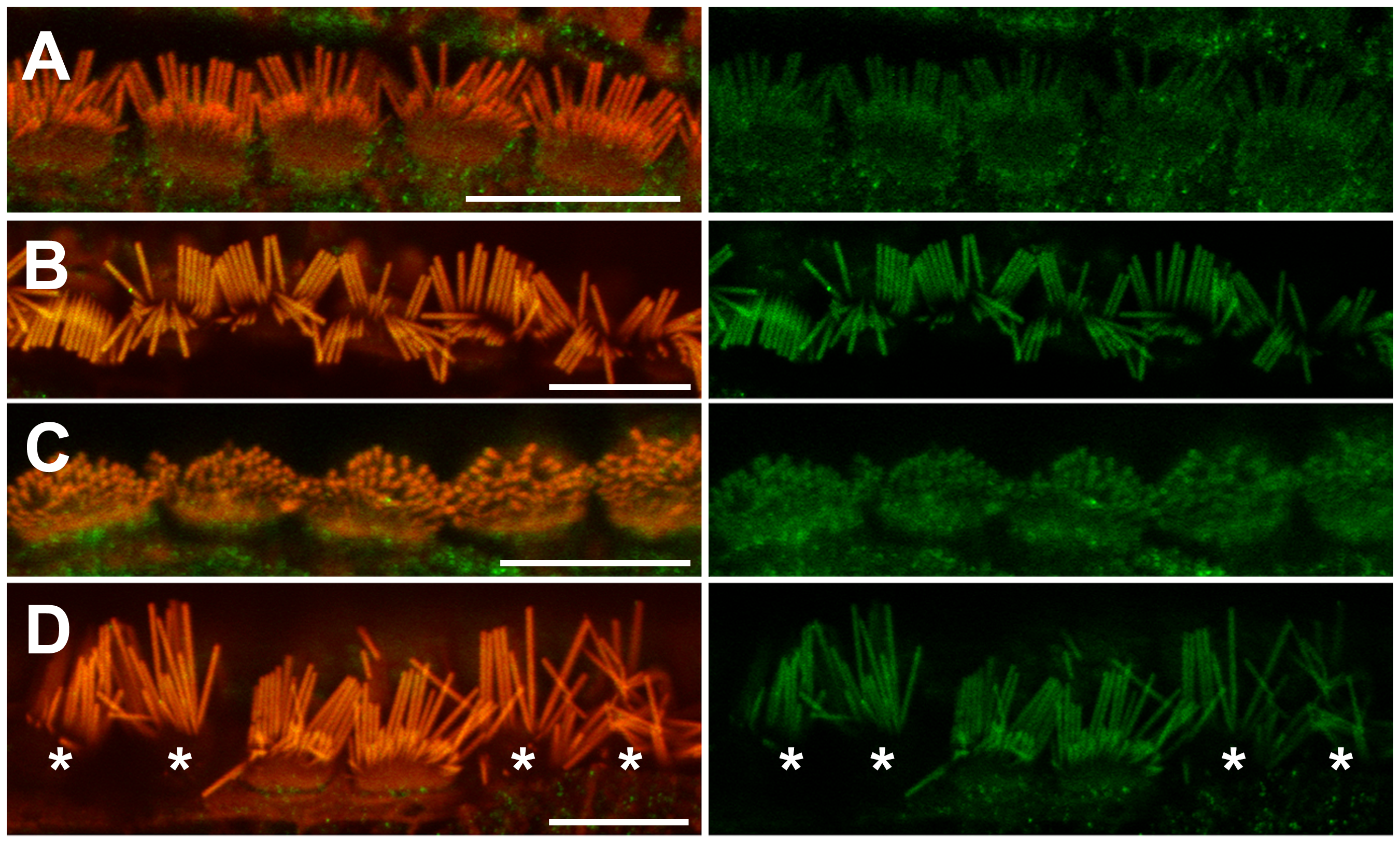

Supplement: Figure S1 — Twinfilin-1 localizes along the length of stereocilia and its immunostaining is not affected by the lack of myosin VIIa or whirlin. Confocal images showing the distribution of twinfilin-1 (green) in stereocilia bundles on the apical surface of inner hair cells. Actin filaments were counterstained with rhodamine/phalloidin. Images on the right show green channel. (A) Wildtype adult mouse, (B) Whrn+/wi adult mouse, (C) Whrnwi/wi adult mouse, (D) The mosaic epithelia of an adult Myo7a4626SB/4626SB Hprt(Myo7a)Brd/+ female (asterisks indicate non-complemented, myosinVIIa-deficient cells). Scale bars: A–D 10 µm. (10.98 MB TIF) [file pone.0007097.s001.tif]

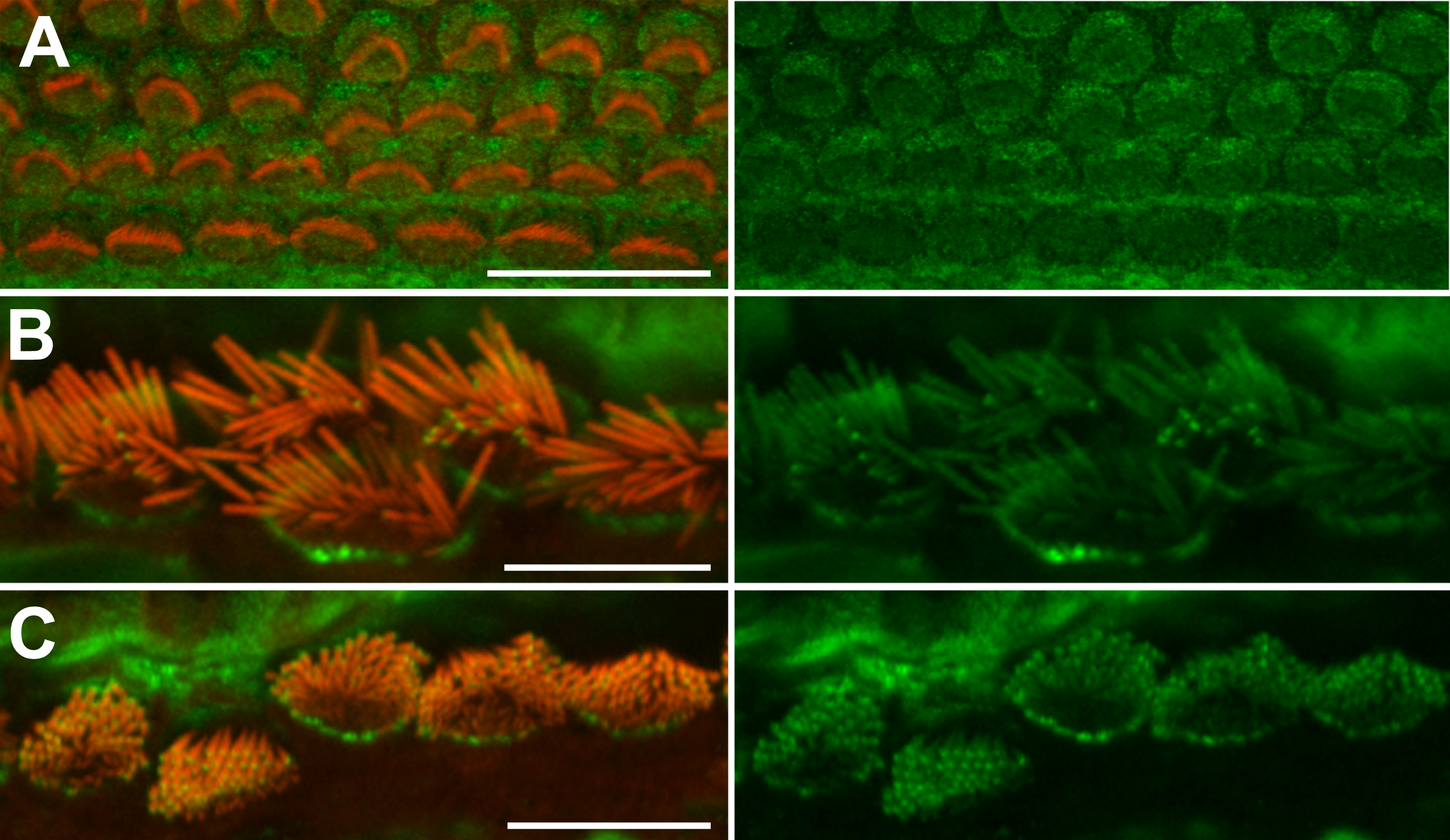

Supplement: Figure S2 — Twinfilin appears in the stereocilia tips between Postnatal Day (PD) 2 and 7 Confocal images showing no pan-twinfilin staining (green) at the tips of control hair cells at PD2 (A). At PD7 pan-twinfilin staining was present at the tips of shorter stereocilia in wild type hair cells (B) and in tips of all stereocilia of Whrnwi/wi mice (C). Scale bars: A 10 µm, B–C 5 µm. (3.21 MB TIF) [file pone.0007097.s002.tif]

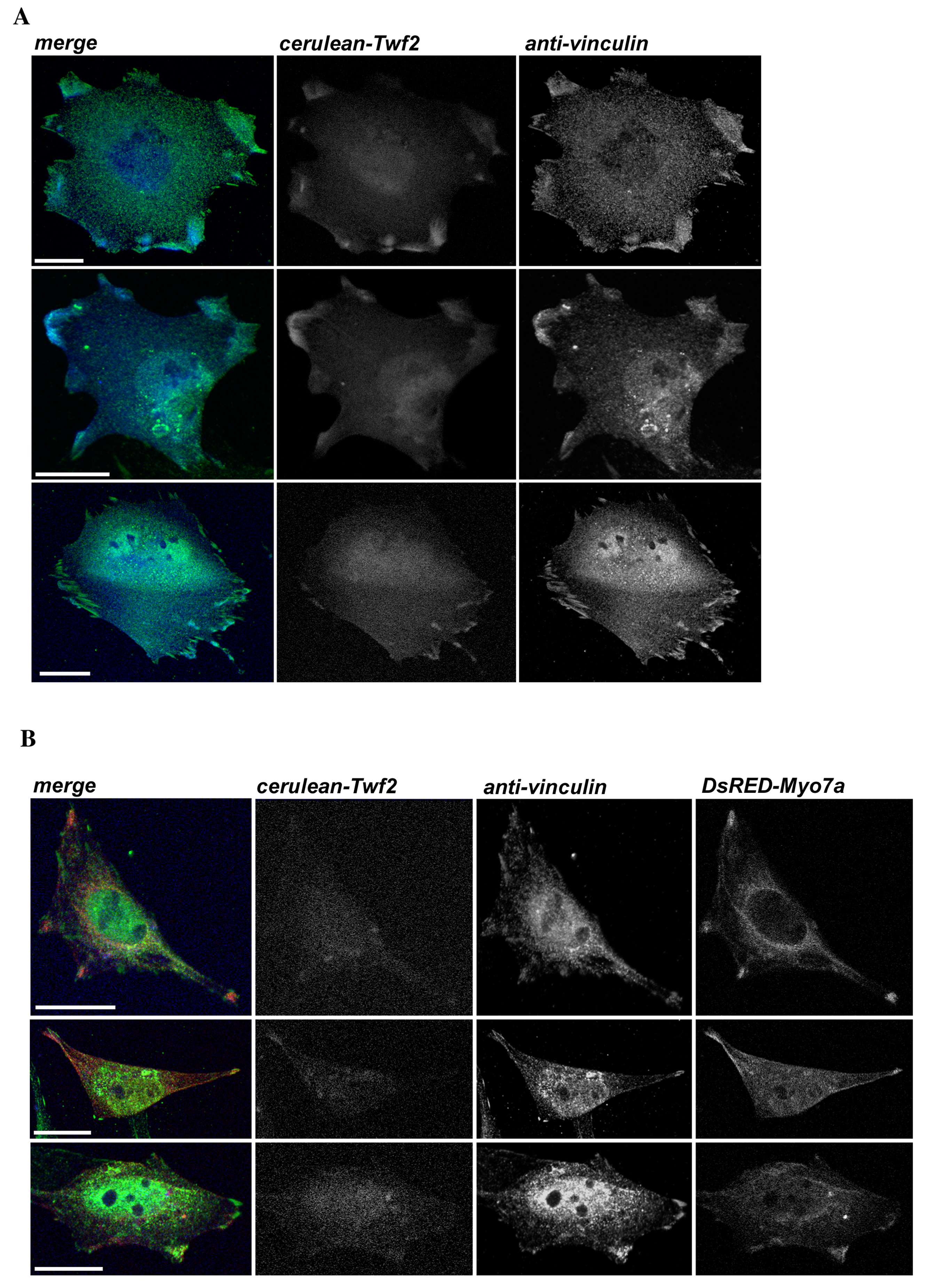

Supplement: Figure S3 — Cerulean-Twf2 and DsRED-Myo7a co-localize with vinculin within focal attachment sites. Confocal images showing BHK-21 fibroblasts transfected with cerulaean-Twf2 alone (A) and co-transfected with cerulean-Twf2 and DsRED-Myo7a (B) and stained with anti-vinculin antibodies. Cerulean-Twf2 and DsRED-Myo7a localize to focal adhesion sites visualized by anti-vinculin immunolabeling. However, in all double transfected cells the Cerulaean-Twf2 signal is very weak and diffuse. Scale bars A,B 20 µm (9.32 MB TIF) [file pone.0007097.s003.tif]
